# DOE analysis of the rheological data of shear-thickening fluids for puncture-resistant composite shells

**DOI:** 10.1016/j.dib.2018.09.107

**Published:** 2018-10-03

**Authors:** Federico Cecchini, Valeria Cherubini, Francesco Fabbrocino, Francesca Nanni

**Affiliations:** a Department of Enterprise Engineering, University of Rome Tor Vergata, Via del Politecnico 1, 00133 Rome, Italy; b Italian Interuniversity Consortium on Materials Science and Technology (INSTM), Research Unit Roma Tor Vergata, Via del Politecnico 1, Rome 00133, Italy; c Department of Civil Engineering, Pegaso University, Italy

## Abstract

The dilatancy (Shear-thickening) is a time-independent rheological behaviour exhibited by some non-Newtonian fluids. These fluids manifest a surge in the apparent viscosity with an increase in the shear rate. If these fluids are encapsulated, they can be used to manufacture high-end stab resistance inserts (Cecchini et al., 2018) [1]. In the first part of this work, a comparison between the rheological performance of different shear-thickening fluids (STFs) based on nanosilica dispersed in glycols is presented. This analysis attempts to investigate the combinations of fillers and carriers with the highest energy-absorbing capabilities, among the various glycol-based colloidal STFs. In the second part, the influence of the composition of the STF on its rheological properties is analyzed statistically. The statistical analysis is performed by evaluating the interaction between the main design variables of the fluids (filler dimension, weight-to-weight ratio and molecular weight of the carrier). Finally, the puncture resistance of a composite system obtained by encapsulating the previously manufactured shear-thickening fluids in a polymeric shell is analyzed by means of a high-speed puncture test. This test is performed on the mockup of a tyre tread containing the best performing STF. The results showed that the use of the STF core increased the resistance to puncture by 20% as compared to the same volume of tyre tread material. Furthermore, the STF hermetically sealed the pierced sample, even when the tyre mockup was inflated at high internal pressures.

## Specifications table

**Table:** 

Subject area	Materials science – Composite materials
More specific subject area	Puncture resistance
Type of data	Tables, graphs
How data was acquired	Rheological tests, Stab tests, Air permeability tests
Data format	Raw data
Experimental factors	The powders were dried in an unventilated oven, to remove excess humidity
Experimental features	The rheological samples were subjected to steady-shear rheometry. The best performing samples were used inside a rubber disk. The rubber disks were subjected to puncture resistance tests, nail extraction and air permeability test.
Data source location	Rome, Italy (Via del Politecnico 1 ,00133)
Data accessibility	Data is with this article
Related research article	[1] F. Cecchini, V. Cherubini, M. Sadaf, F. Fabbrocino, F. Nanni, Design of a puncture-resistant composite shell comprising a non-Newtonian core, Polym. Test. 67 (2018) 494–502. 〈http://dx.doi.org/10.1016/j.polymertesting.2018.03.019〉.
	[2] F. Cecchini, V. Cherubini, M. Sadaf, F. Fabbrocino, F. Nanni, Design of a puncture-resistant composite shell comprising a non-Newtonian core, Polym. Test. 67 (2018) 494–502. 〈http://dx.doi.org/10.1016/j.polymertesting.2018.03.019〉.
	[3] T.A. Hassan, V.K. Rangari, S. Jeelani, Sonochemical synthesis and rheological properties of shear thickening silica dispersions., Ultrason. Sonochem. 17 (2010) 947–952. 〈http://dx.doi.org/10.1016/j.ultsonch.2010.02.001〉.
	[4] M. Hasanzadeh, V. Mottaghitalab, M. Rezaei, Rheological and viscoelastic behavior of concentrated colloidal suspensions of silica nanoparticles: A response surface methodology approach, Adv. POWDER Technol. (2015). 〈http://dx.doi.org/10.1016/j.apt.2015.08.011〉.
	[5] F. Cecchini, V. Cherubini, F. Nanni, A D.O.E. analysis of the rheological properties of non-Newtonian fluids with different compositions, in: ECNP IX, 2016.

## Value of the data

● The data included in this article can be used for the preliminary evaluation of the stab resistance of composite shells comprising a Shear-Thickening core.
● In particular, during the first part of the article a statistical analysis of the effect of the composition of nanocomposite shear-thickening fluids (STFs) to their rheological response is performed.
● During the second part, a statistical analysis of the effect of the composition of nanocomposite shear-thickening fluids to the repeatability of their rheological response.
● This analysis may prove useful for the Large-Scale Production of these materials.
● Finally, in order to the aforementioned results, the best performing Shear-thickening fluid is used for the design of a concept for a novel tyre tread with increased puncture resistance and with self-sealing properties and the prototype is subjected to an instrumented stab-test.

## 1 Data

**Fig. 1:**
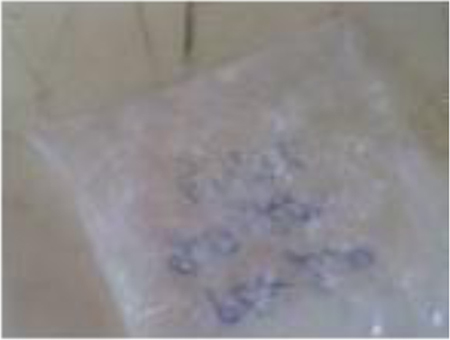
Example of an STF sample used for the rheological analysis.

**Fig. 2:**
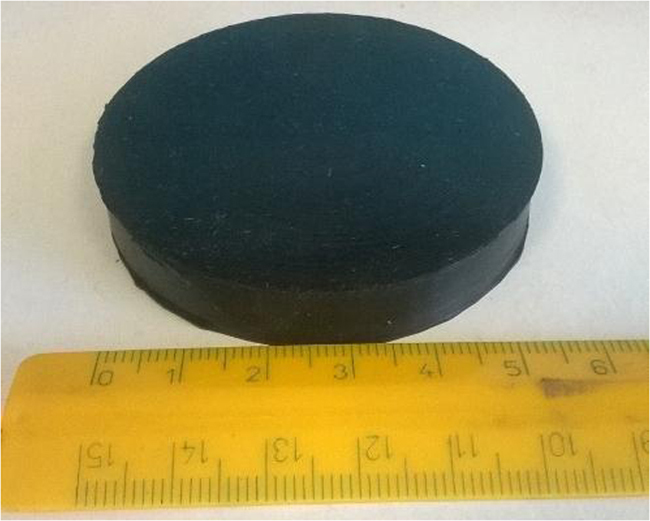
Sketch of the penetration resistance sample.

**Fig. 3:**
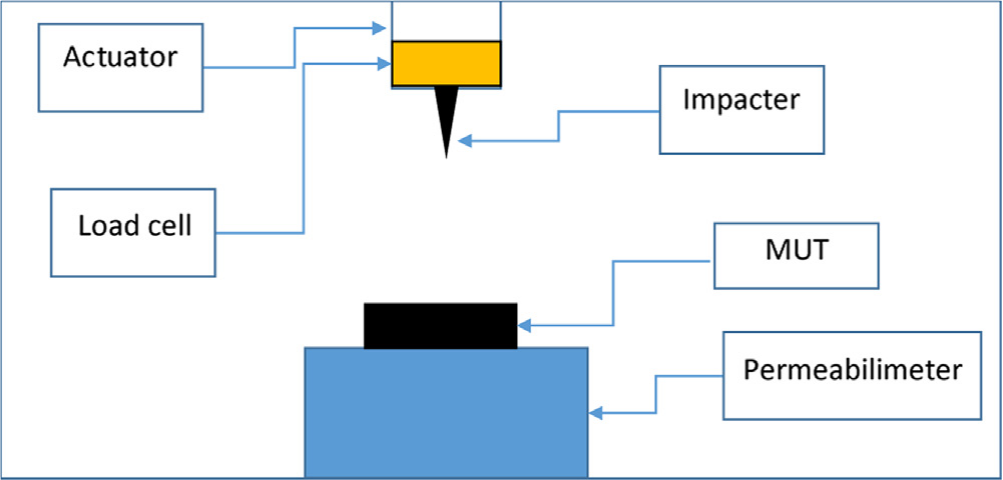
Sketch of the penetration resistance apparatus.
• Fig. 4: interaction plot relating the influence of the chemical composition of the samples to the figure of merit “increase in the apparent viscosity” Δ*η*.

The data presented in this article is supplementary to our research [1], [2] and consists of:

• Table 1: summarizing the results of the Steady-shear rheological analysis performed on the STFs samples. In this table, the main parameters useful for the design of stab resistant inserts (i.e. increase in the apparent viscosity after the impact and critical shear rate) are listed (Fig. 1, Fig. 2, Fig. 3).

**Table 1:** Rheological results of the best-performing formulations. The results are averaged values of ten repeated tests.

**Type of silica**	**Average particle diameter (nm)**	**Silica content (wt%)**	**Matrices**	
			**Peg (MW)**	
			**200**	**400**
Fumed (Sigma-Aldrich)	12	20	Peg200/20Fs	Peg400/20Fs
			Δ*η* = 298,5 ± 9.7 Pa*s	Δ*η* =58.0 ± 0.9 Pa*s
			${\dot{\gamma }}_{c}$**= 30.0 ± 0.9 s**^**-1**^	${\dot{\gamma }}_{c}$**= 30.0 ± 0.9 s**^**-1**^
		30	Peg200/30Fs	Peg400/30Fs
			Δ*η* = 1985.0 ± 69.1 Pa*s	Δ*η* = 580 ± 21.7 Pa*s
			${\dot{\gamma }}_{c}$**= 18 ± 0.6 s**^**-1**^	${\dot{\gamma }}_{c}$**= 10.0 ± 0.4 s**^**-1**^
Fumed (Sigma-Aldrich)	200	20	Peg200/20Fb	Peg400/20Fb
			Δ*η* = 58.5 ± 2.7 Pa*s	Δ*η* = 25.0 ± 2.7 Pa*s
			${\dot{\gamma }}_{c}$**= 62.0 ± 2.8 s**^**-1**^	${\dot{\gamma }}_{c}$**= 250.0 ± 8.8 s**^**-1**^
		30	Peg200/30Fb	Peg400/30Fb
			Δ*η* = 496± 19.9 Pa*s	Δ*η* = 180 ± 6.3 Pa*s
			${\dot{\gamma }}_{c}$**= 40.0 ± 1.6 s**^**-1**^	${\dot{\gamma }}_{c}$**= 60.0 ± 2.1 s**^**-1**^

**Fig. 4:**
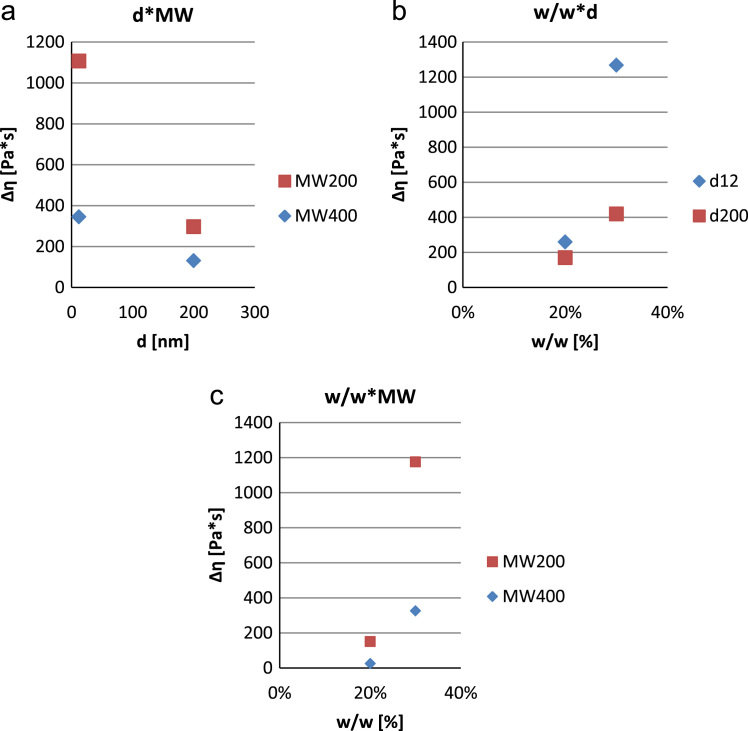
Interaction plot for ∆η (a–c).
• Fig. 5: interaction plot relating the influence of the chemical composition of the samples to the figure of merit “critical shear rate” ${\dot{\gamma }}_{c}$.

**Fig. 5:**
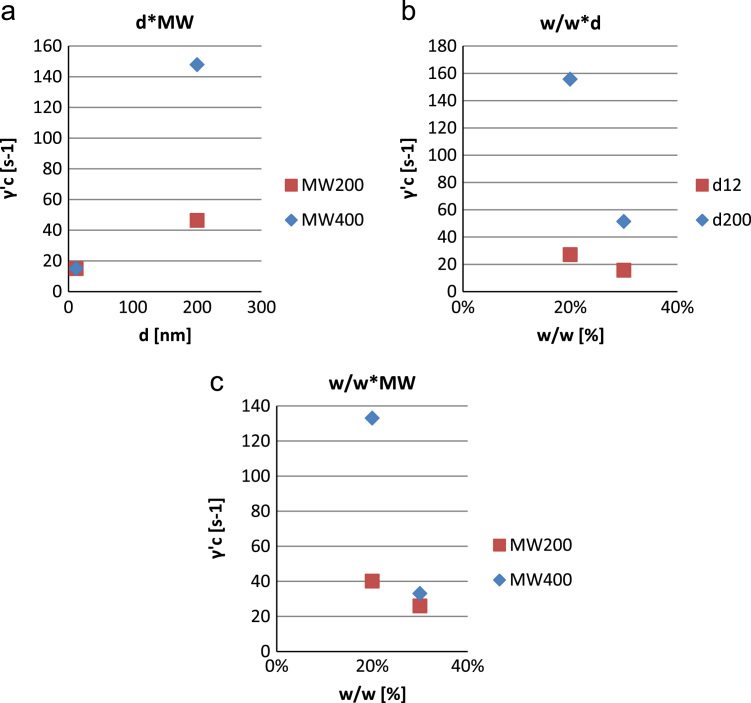
Interaction plot for ${\dot{\gamma }}_{c}$ (a–c).
• Fig. 6: interaction plot relating the influence of the chemical composition of the samples to the standard deviation of the figure of merit “increase in the apparent viscosity” Δ*η*.

**Fig. 6:**
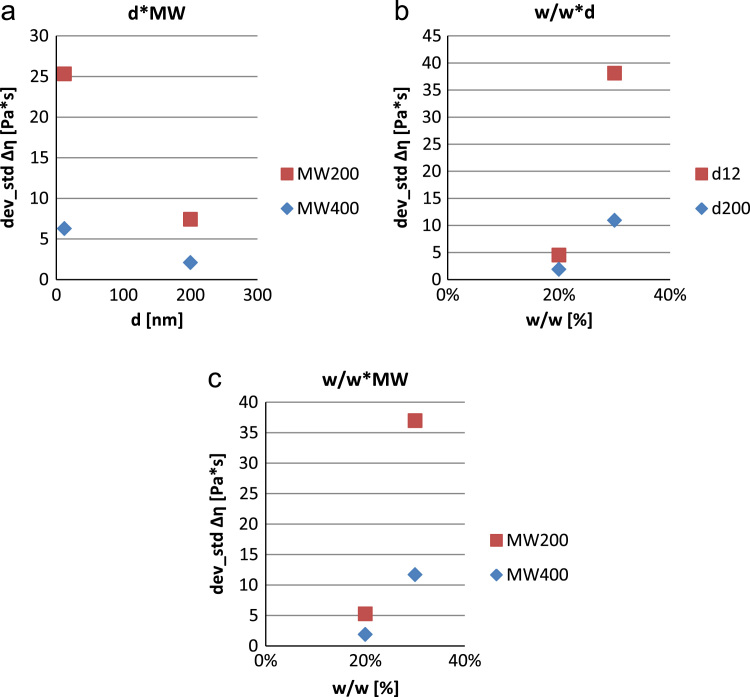
Interaction plot for the standard deviation of ∆η (a–c).
• Fig. 7: interaction plot relating the influence of the chemical composition of the samples to the standard deviation of the figure of merit “critical shear rate” ${\dot{\gamma }}_{c}$.

**Fig. 7:**
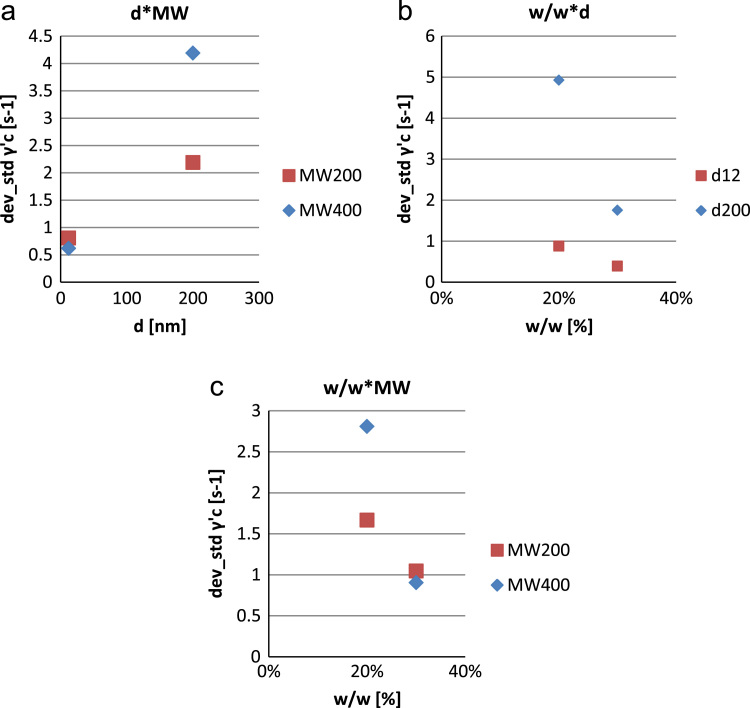
Interaction plot for the standard deviation of ${\dot{\gamma }}_{c}$ (b).
• Fig. 8 Force–displacement curves measuring the puncture resistance and the nail extraction resistance of the rubber shell for the best performing samples.

**Fig. 8:**
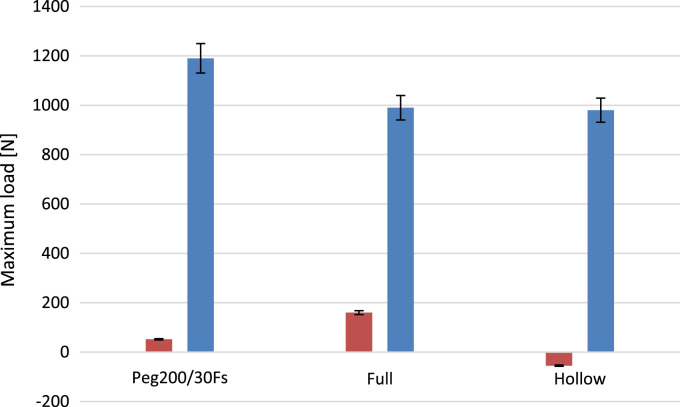
Maximum load during puncture and extraction for the various samples.
• Fig. 9 Air permeability test data (time vs pressure) performed on the punctured samples for the best performing samples.

**Fig. 9:**
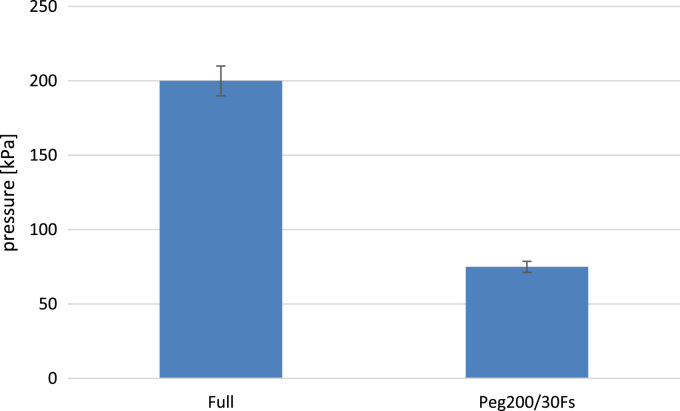
Pressure inside the chamber 1 s after nail removal.

## 2 Experimental design, materials, and methods

### 2.1 Materials

Three different polymers with different molecular weights were chosen as carrier fluids:

• Polyethylene glycol (Peg, Sigma-Aldrich, MW 200, 400, 1000).
• Polypropylene glycol (Ppg, Sigma-Aldrich, MW 425, 725, 1000).
• Ethylene glycol (Eg, Carlo Erba, Analytical Standard MW 62.07).

The filler chosen was silica with different shapes and average particle dimension:

• Spherical fumed silica (Sigma-Aldrich, average diameter *d* = 12 nm).
• Spherical fumed silica (Evonik R104, average diameter *d* = 7 nm).
• Irregularly shaped fumed silica (AEROSIL, Sigma-Aldrich, average diameter *d* = 12 nm).
• Irregularly shaped fumed silica (AEROSIL, Sigma-Aldrich, average diameter *d* = 200 nm).

## 3 Methods

### 3.1 Rheological tests

The nanopowders were preliminarily dried in an unventilated oven for 8 h at 50 °C, dispersed in ethanol ({weight of ethanol}:{weight of filler} = 7.9:6.5) and then the polymeric matrix was added and the slurry was vigorously jar-stirred approximately 1 h per 50 ml. At the end of the process, the colloid was placed in an ultrasound bath for 1 h at 80 °C, to remove the remaining ethanol [3].

The rheological experimental campaign was planned according to a generalized full factorial design with five factors:

• Carrier fluid (three levels).
• Molecular weight of the carrier (three levels per fluid, except for Eg).
• Shape of the filler (two levels).
• Dimension of the filler (two levels per shape).
• Weight-to-weight ratio of filler to carrier (two levels, 20% and 30%).

The total number of different samples (Fig. 1) analyzed during the experimental campaign was 63 (56 colloids plus the unfilled 7 carrier fluids used as a reference).

For every combination, a set of ten samples of each type was tested by means of steady-shear rheometry (Malvern Kinexus Lab+ strain-controlled rheometer) with the following parameters [4]:

• Fixture: conical (diameter = 25 mm, angle = 0.1 rad).
• equilibration step: 2 min at 1 s^-1^.
• Shear rate $\dot{\gamma }$ range: [1E-1; 1E4] s^-1^. A conical fixture was used in order to achieve a uniform shear rate along the radius.
• Test temperature: 25 °C.

### 3.2 Penetration resistance test samples

The penetration resistance samples (MUT) were manufactured by stacking and then cross-linking three disks (diameter = 4.5 cm, thickness = 0.5 cm) of a styrene-butadiene rubber (SBR) based compound. The central disk contained a circular hole (diameter = 1.5 cm) and was completely filled with the best performing STF (Fig. 2).

The high-speed puncture tests were based on ISO 6603, with the nail connected to an actuator moving at a prescribed high speed (500 mm/s). The testing actuator has an integrated load cell capable of measuring both the load necessary to pierce the MUT and that necessary to remove the nail (Fig. 3).

The MUT is positioned at the top of a pressurized vessel as the closure cap. The vessel is connected to an air permeability tester (max pressure = 200 kPa). During the test, the effective pressure inside the chamber is measured by means of an integrated pressure gauge.

### 3.3 Steady-shear rheometry

The figures of merit chosen were the increase in the apparent viscosity of the fluid (Δη) and the critical shear rate (${\dot{\gamma }}_{c}$) associated with the Shear-thickening effect [5]. The higher the Δ*η*, the higher the energy absorbed under impact. Regardless of the particle diameter, AEROSIL dispersed in Polyethylene glycol proved to be the best performing sample (data for the best performing samples collected in Table 1).

The influence of the previously listed control parameters, as well as interactions of them up to the second order, can be analyzed by looking at the interaction plots. By comparing the charts, from a merely phenomenological point of view and with a 95% confidence, it can be clearly seen that different parameters have am higher influence on the fluid׳s rheological response in terms of ∆η and ${\dot{\gamma }}_{c}$.

In particular, w/w and φ are the most influential parameters to the rheological response of the fluid, both in terms of ∆η and ${\dot{\gamma }}_{c}$ whereas MW is less significant (Figs. 4 and 5).

The previous results can be justified by considering that the surge in the dilatant effect is enabled by the formation of complex aggregates of particles known as hydroclusters. As a matter of fact, a high number of smaller particles dispersed in a low molecular weight carrier (i.e. less viscous) can form more easily hydroclusters. The higher the number of hydroclusters, the more remarkable the dilatancy in the Shear-thickening fluid (higher ∆η and lower ${\dot{\gamma }}_{c}$).

The repeatability of tests (10 for each sample) was verified by analysis of the standard deviations of the results obtained for ∆η and ${\dot{\gamma }}_{c}$ (Figs. 6 and 7).

Contrarily to what previously shown for ∆η and ${\dot{\gamma }}_{c}$, from a phenomenological point of view and with a 95% confidence, as far as the standard deviation of ∆η is concerned only w/w is relevant while φ is the most relevant to the standard deviation of ${\dot{\gamma }}_{c}$. The other parameters and higher order effects are negligible.

### 3.4 Puncture resistance of a mock-up of a tyre tread

The SBR puck with the best performing STF was subjected to high-speed puncture tests.

The results for the filled sample, an empty shell and a full SBR puck are compared in Fig. 8.

Furthermore, the STF proved to seal the hole (Fig. 9) after the nail removal contrarily to the reference samples (Hollow disk and Full disk). This can be explained physically by noticing that the fluid in the hole undergoes sever shear stresses and, as a consequence, the dilatancy activates and the fluid cannot flow.
